# A Solid-State Fluorescence Switch Based on Triphenylethene-Functionalized Dithienylethene With Aggregation-Induced Emission

**DOI:** 10.3389/fchem.2021.665880

**Published:** 2021-04-28

**Authors:** Haining Zhang, Xiaoxiao Hu, Huijuan Zhu, Limin Shen, Congmin Liu, Xiaoman Zhang, Xinyu Gao, Lingmei Li, Yan-Ping Zhu, Ziyong Li

**Affiliations:** ^1^Luoyang Key Laboratory of Organic Functional Molecules, College of Food and Drug, Luoyang Normal University, Luoyang, China; ^2^Key Laboratory of Molecular Pharmacology and Drug Evaluation, Ministry of Education, Collaborative Innovation Center of Advanced Drug Delivery System and Biotech Drugs in Universities of Shandong, School of Pharmacy, Yantai University, Yantai, China

**Keywords:** dithienylethene, fluorescence switch, aggregation-induced emission, photochromism, triphenylethene

## Abstract

The development of novel dithienylethene-based fluorescence switches in the aggregated state, and the solid state is highly desirable for potential application in the fields of optoelectronics and photopharmacology. In this contribution, three novel triphenylethene-functionalized dithienylethenes **(1–3)** have been designed and prepared by appending triphenylethene moieties at one end of dithienylethene unit. Their chemical structures are confirmed by ^1^H NMR, ^13^C NMR, and HRMS (ESI). They display good photochromic behaviors with excellent fatigue resistance upon irradiation with UV or visible light in Tetrahydrofuran (THF) solution. Before irradiation with UV light, they exhibit Aggregation Induced Emission (AIE) properties and luminescence behaviors in the solid state. Moreover, upon alternating irradiation with UV/visible light, they display effective fluorescent switching behaviors in the aggregated state and the solid state. The experimental results have been validated by the Density Functional Theory (DFT) calculations. Thus, they can be utilized as novel fluorescence switches integrated in smart, solid-state optoelectronic materials and photopharmacology.

**Graphical Abstract:**
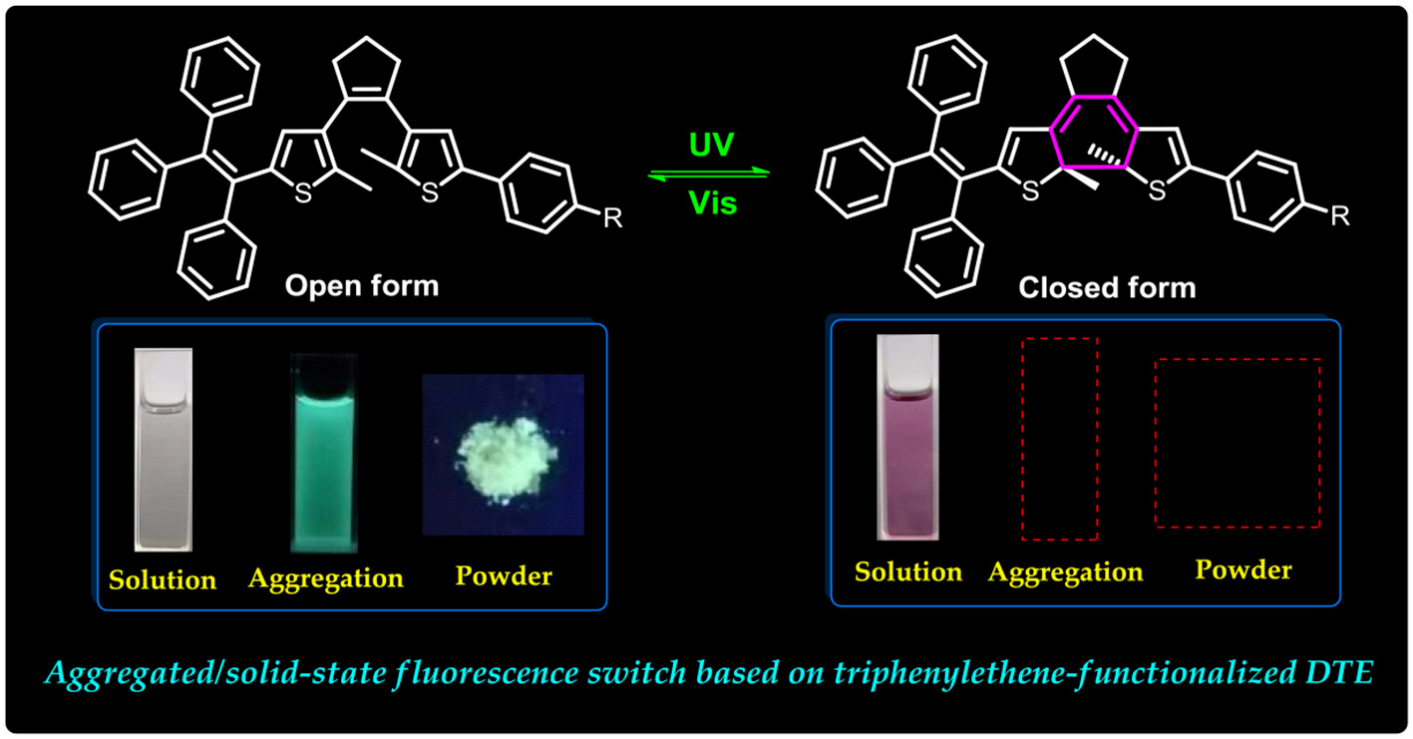
They displayed effective fluorescent switching behaviors in the aggregated state and solid state.

## Introduction

In recent years, fluorescence switches have received increasing attention due to their potential applications in super-resolution fluorescence microscopies and optical data storage (Irie et al., 2002; Qiang et al., 2018; Yu et al., 2018). Generally, the elaboration of switch systems combining photochromic unit and fluorescence groups can be modulated with optical stimulations through intramolecular energy/electron transfer (Raymo and Tomasulo, 2005). Dithienylethene (DTE), a family of classic P-type-photochromic compounds, can be reversibly transformed between ring-open and ring-closed isomers by photoirradiation, which is appealing for optical switching of fluorescence on account of high thermal stability, rapid response, and fatigue resistance (Irie, 2000; Irie et al., 2000, 2014; Tian and Yang, 2004; Zhang et al., 2014; Pu et al., 2016; Yao et al., 2016; Lubbe et al., 2017; Zhang and Tian, 2018; Li et al., 2019a; Li Z. et al., 2020). In recent years, great progress has been made in the fluorescence switches-based dithienylethene unit (Myles et al., 2010; Fukaminato et al., 2011; Uno et al., 2011; Li et al., 2014; Yao et al., 2016). However, most normal fluorophores suffer from fluorescence weakening or quenching at high concentration or in the aggregated state, which is known as “aggregation-caused quenching” (ACQ) caused by the strong intermolecular π**–**π interaction or hydrogen bonding between neighboring fluorophores (Cui et al., 2016; Ma et al., 2018; Zhou et al., 2020), thus limiting applications of these photoswitches in the optoelectronics and photopharmacology in the future. Therefore, it will be highly desirable to develop the aggregated/solid-state fluorescence switches for potential applications.

**Scheme 1:**
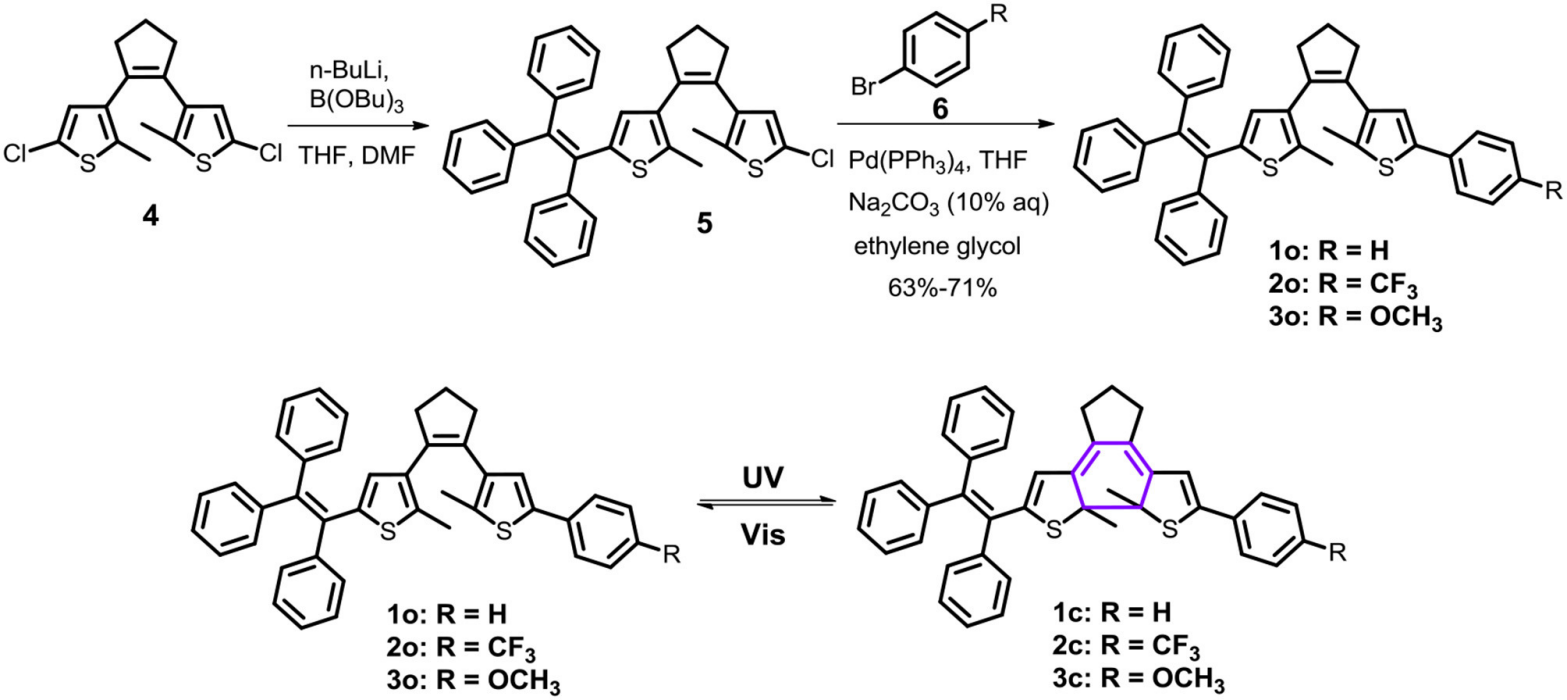
Synthetic route and photochromism of dithienylethenes **1**–**3**.

Fortunately, Tang's group discovered a novel class of fluorophores with aggregation-induced emission (AIE) in 2001 (Luo et al., 2001), which is opposite to the conventional ACQ phenomenon. This interesting phenomenon provides a new direction to design organic fluorescent materials with more widely and greater practical applications in the aggregated state or the solid state (Ding et al., 2013; Mei et al., 2014; Liang et al., 2015; Li et al., 2017; Li H. et al., 2020; Li X. et al., 2020; Tian et al., 2020; Zang et al., 2021). To the best of our knowledge, the most simplest approach to achieve the aggregated/solid-state fluorescence switches is combining the photochromic reaction of DTE and fluorescence of solid emitters (such as naphthalimide (Wang et al., 2006; Jiang et al., 2007, 2009), perylene bisimide (Fukaminato et al., 2011; Berberich et al., 2012; Li et al., 2014), tetraphenylethene (Li et al., 2013; Dong et al., 2016; Ma et al., 2020), and cyano-substituted ethylene (Lim et al., 2004, 2005; Wang et al., 2018) to afford high-contrast fluorescence switches in the aggregated or solid state. Recently, our group has developed a novel, solid-state fluorescence switch triggered by blue light (460–470 nm) and NIR light (7,600–770 nm), in which carbazole and BF_2_bdk moieties are suspended on both sides of the dithienylethene unit (Li et al., 2019c). However, we still know very little about such aggregated/solid-state fluorescence switches. Consequently, it is urgently necessary to develop novel DTE-based fluorescence switches in the aggregated state and the solid state for the practical application requirements. In addition to the tetraphenylethene (TPE), the more readily available triphenylethene (TriPE) is also a typical aggregation-induced-emission active group. Herein, we have developed three novel triphenylethene-functionalized dithienylethenes (**1**–**3**), as shown in Scheme 1. And their photochromism, AIE properties, and fluorescent switching behaviors in the aggregated state and the solid state have been thoroughly investigated.

## Materials and Methods

### Materials

Manipulation is carried out under a nitrogen atmosphere, using standard Schlenk techniques unless otherwise stated. THF was distilled under nitrogen from sodium-benzophenone. The intermediates **4** (Lucas et al., 2003) and **5** (Dong et al., 2016) are prepared by reported literature methods. All other starting materials are obtained commercially as analytical-grade and used without further purification. The cyclization and cycloreversion quantum yields of dithienylethenes **1**–**3** are determined by comparing the reaction yield with the known yield of the compound 2-bis(2-methyl-5-phenyl-3-thienyl)perfluorocyclopentene (Irie et al., 2000).

### Instruments

^1^H and ^13^C NMR spectra are collected on German BRUKER AVANCE III 400 MHz (all the chemical shifts are relative to TMS). High-resolution mass spectra are obtained on SCIEX X-500R QTOF (ESI mode). All the absorption spectra are collected on a SHIMADZU UV-2600 UV-Vis spectrophotometer. In the photochromic experiments, UV light irradiation (254 nm) is carried out, using a ZF5UV lamp; and visible light is irradiated, using an LZG 220 V 500 W tungsten lamp (λ > 402 nm) with cut-off filters.

### Synthesis of Dithienylethenes 1–3

To a solution of **5** (548 mg, 1 mmol) in anhydrous THF (10 ml), *n*-BuLi (0.4 ml of 2.5 M solution in hexane, 1 mmol) is slowly added under N_2_ in an ice bath and stirred for 1 h at 0°C. Then B(OBu)_3_ (0.41 ml, 1.5 mmol) is added to the above solution and stirred for 6 h at room temperature. Then, the resultant reddish solution is added dropwise to a solution, containing bromobenzene (156 mg, 1 mmol), Pd(PPh_3_)_4_ (25 mg, 0.02 mmol) in THF (10 ml) and Na_2_CO_3_ (2 M, 10 ml) at 60°C. The mixture is refluxed for 16 h under N_2_. The reaction solution is cooled to room temperature and extracted with ethyl acetate (3 × 20 ml), and the combined organic layer is washed with the saturated brine (2 × 20 ml). The organic layer is dried over Na_2_SO_4_, filtered and concentrated under reduced pressure. The residue is purified by column chromatography (silica gel: 200**–**300, PE) to afford dithienylethene **1** as a light yellow solid (Yield: 66%). ^1^H NMR (400 MHz, CDCl_3_) δ 7.49 (d, *J* = 7.6 Hz, 2H), 7.35 (t, *J* = 7.6 Hz, 2H), 7.23 (br, 6H), 7.11**–**7.08 (m, 5H), 7.03**–**7.01 (m, 3H), 6.96**–**6.93 (m, 3H), 6.28 (s, 1H), 2.73 (t, *J* = 6.3 Hz, 2H), 2.54 (d, *J* = 6.4 Hz, 2H), 1.98**–**1.94 (m, 2H), 1.92 (s, 3H), 1.78 (s, 3H). ^13^C NMR (100 MHz, CDCl_3_) δ 143.69, 143.28, 142.93, 141.76, 139.98, 139.42, 136.66, 135.65, 134.95, 134.7, 134.57, 134.21, 134.04, 133.99, 131.24, 130.97, 130.94, 130.8, 128.73, 128.2, 127.56, 127.44, 126.97, 126.89, 126.75, 126.13, 125.29, 124.04, 38.36, 38.31, 22.86, 14.56, 14.16.HRMS (ESI-TOF) m/z: [M + H]^+^Calcd. for C_41_H_35_${\text{S}}_{2}^{+}$591.218; found 591.2152.

Dithienylethene **2** is synthesized by an analogous method to dithienylethene **1** as a yellow solid (yield: 71%). ^1^H NMR (400 MHz, CDCl_3_) δ 7.61**–**7.56 (m, 3H), 7.24 (br, 6H), 7.12**–**7.07 (m, 5H), 7.01 (br, 4H), 6.95**–**6.93 (m, 2H), 6.26 (s, 1H), 2.73 (t, *J* = 7.2 Hz, 2H), 2.56 (t, *J* = 7.2 Hz, 2H), 1.99**–**1.91 (m, 2H), 1.94 (s, 3H), 1.78 (s, 3H). ^13^C NMR (100 MHz, CDCl_3_) δ 143.84, 143.39, 143.08, 142.09, 140.29, 138.09, 137.81, 137.29, 136.06, 135.80, 135.44, 134.99, 134.23, 134.13, 133.77, 131.38, 131.09, 130.97, 128.92, 128.39, 127.75, 127.64, 127.18, 126.95, 126.35, 125.94, 125.9, 125.67, 125.39, 38.49, 38.45, 23.01, 14.77, 14.31.HRMS (ESI-TOF) m/z: [M + H]^+^Calcd. for C_42_H_34_F_3_${\text{S}}_{2}^{+}$659.2054; found 659.2031.

Dithienylethene**3** is synthesized by an analogous method to dithienylethene **1** as a yellow solid (yield: 63%). ^1^H NMR (400 MHz, CDCl_3_) δ 7.42 (d, *J* = 8.7 Hz, 2H), 7.25 (br, 5H), 7.12**–**7.08 (m, 5H), 7.02 (br, 3H), 6.95**–**6.88 (m, 4H), 6.82 (s, 1H), 6.28 (s, 1H), 3.83 (s, 3H), 2.72 (t, *J* = 7.2 Hz, 2H), 2.55 (t, *J* = 7.1 Hz, 2H), 1.98**–**1.92 (m, 2H), 1.90 (s, 3H), 1.77 (s, 3H). ^13^C NMR (100 MHz, CDCl_3_) δ 158.69, 143.66, 143.26, 142.89, 141.65, 139.85, 139.26, 136.47, 135.6, 134.98, 134.46, 134.03, 133.99, 133.12, 131.25, 131, 130.95, 130.8, 128.2, 127.55, 127.43, 126.97, 126.75, 126.52, 126.11, 122.89, 114.09, 55.34, 38.34, 38.26, 22.8, 14.5, 14.18. HRMS (ESI-TOF) m/z: [M + H]^+^Calcd. for C_42_H_37_${\text{OS}}_{2}^{+}$621.2286; found 621.2279.

## Results and Discussions

### Photochromic Properties in Solution

**Figure 1:**
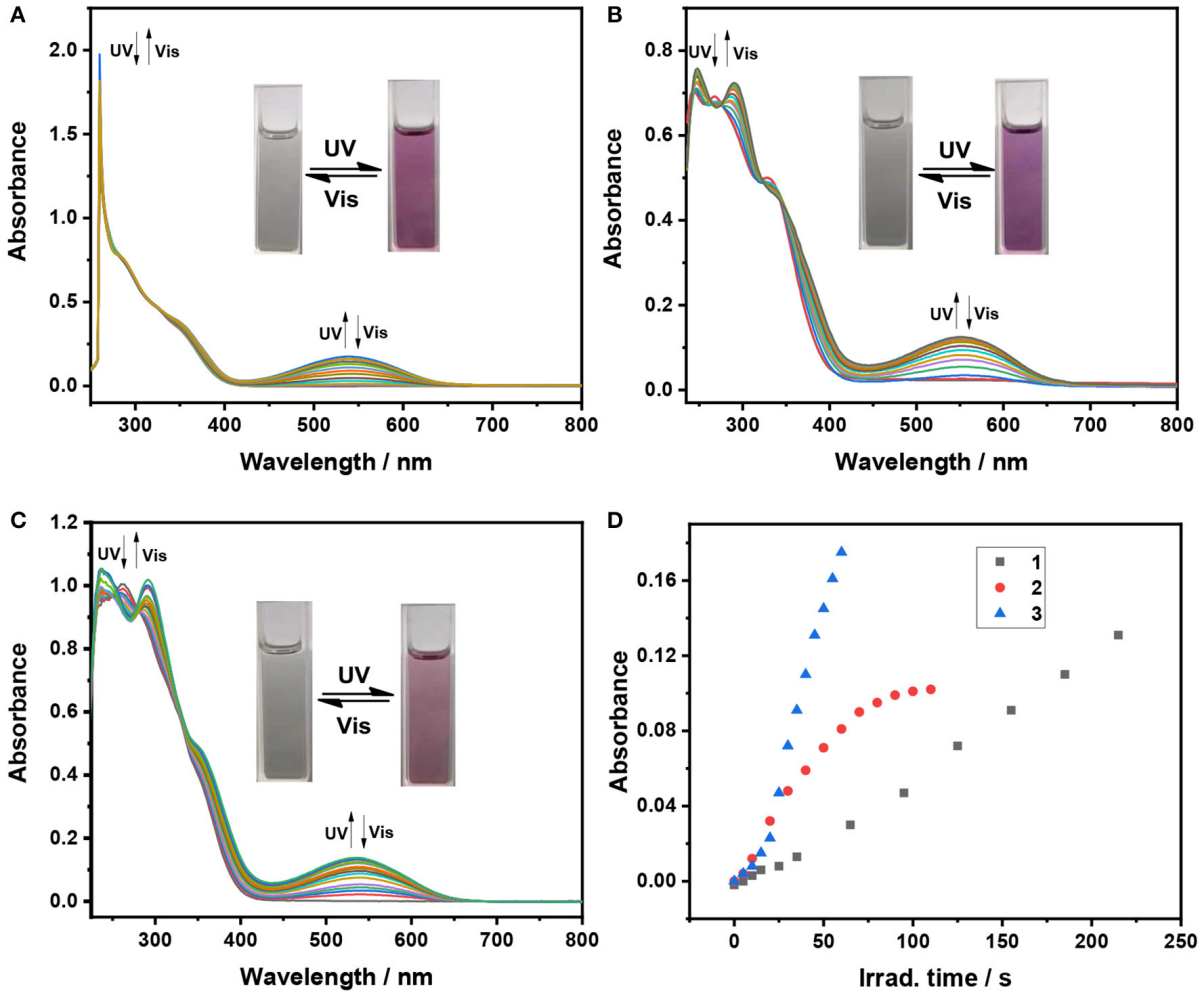
Absorption spectral changes of dithienylethenes **1–3** with 254 nm UV and >402 nm Vis light irradiation in THF (2 × 10^−5^ mol/L), **(A)** spectral changes for **1** (0–395 s for cyclization, irradiation interval: 5 s; 0–650 s for cycloreversion, irradiation interval: 10 s); **(B)** spectral changes for **2** (0–110 s for cyclization, irradiation interval: 5 s; 0–195 s for cycloreversion, irradiation interval: 10 s); **(C)** spectral changes for **3** (0–85 s for cyclization, irradiation interval: 5 s; 0–125 s for cycloreversion, irradiation interval: 10 s); **(D)** the optical response rate monitored at the maximum absorption wavelength in the visible region for ring-closed isomers **1c**–**3c**.

Firstly, photochromic properties of the triphenylethene-functionalized dithienylethene **1–3** are investigated upon alternating irradiation with 254 nm UV light and visible light (>402 nm) in THF, which undergo photoisomerization between the open form and the closed form (Scheme 1). As depicted in Figure 1A, the absorption maximum of ring-open isomer **1 (o)** in THF is observed at 260 nm (ε = 6.58 × 10^4^ M^−1^cm^−1^) as a result of a π**–**π^*^ transition (Li et al., 2008). Upon irradiation with 254 nm UV light, a new absorption band at 540 nm (ε = 0.81 × 10^4^ M^−1^cm^−1^) appears along with an obvious color change from colorless to pink as a result of the formation of the corresponding ring-closed isomer **1 (c)** (Scheme 1). Moreover, an obvious isosbestic point that appears at 323 nm is observed, which indicates a clean photochemical transformation between the open isomer **1 (o)** and closed isomer**1 (c)**because isosbestic point generally means the coexistence of both open and closed forms. Upon irradiation with >402 nm visible light, the pink closed isomer **1 (c)** performs a cycloreversion reaction to form the initial colorless open isomer. Particularly, good reversibility for photochromism can be observed upon alternating photoirradiation into the ring-open and ring-closed isomers of dithienylethene **1** (Supplementary Figure 1). The cyclization and cycloreversion quantum yields of **1** in THF are.13 (ϕ_o−c_) and 0.0074 (ϕ_c−o_) (Table 1), respectively.

**Table 1:** Photochromic parameters of dithienylethenes **1**–**3** in THF (2 × 10^−5^ M) and emission data in the aggregated and powder states.

**Compounds**	**λ_max_^a^ (nm) (ε × 10^**4**^, M^−1^cm^−1^)**	**λ_max_^b^ (nm) (ε × 10^**4**^, M^−1^cm^−1^)**	**ϕ_o-c_^c^**	**ϕ_c-o_^d^**	**λ_em_^e^ (nm)**	**λ_em_^f^ (nm)**
	(Open)	(PSS)				
**1**	260 (6.58)	540 (0.81)	0.13	0.0074	495	493
**2**	268 (2.51)	552 (0.63)	0.25	0.0079	491	487
**3**	262 (5.03)	538 (0.69)	0.34	0.0084	494	490

a *Absorption maxima of ring-open isomers*.

b *Absorption maxima of ring-closed isomers*.

c *The cyclization quantum yields (ϕ_c−o_)*.

d *The cycloreversion quantum yields (ϕ_o−c_)*.

e *Emission maxima of ring-open isomers in the aggregated state*.

f *Emission maxima of ring-open isomers in the powder state, respectively*.

Similar photochromic behaviors are observed when THF solutions of **2** and **3** are exposed to 254 nm UV light and >402 nm visible light, respectively, as illustrated in Figures 1B,C, Supplementary Figures 2, 3. Moreover, their optical response rates into luene are sequenced in the following order in **3** > **2** > **1** (Figure 1D), implying that **3** and **2** can achieve the photo stationary state more efficiently than analog **1** without substitution. The data from Table 1 revealed that different substituent groups have a slight effect on their photochromic properties, mainly including the absorption maximum and quantum yields of cyclization and cycloreversion reactions. For dithienylethene **2** with the trifluoromethyl group, the maximum absorption wavelengths of ring-open isomer [268 nm for **2 (o)**] and ring-closed isomer [552 nm for **2 (c)**] display a distinct bathochromic shift compared with those of **1a** without substitution and **3** with the OCH_3_ group, which can be attributed to the fact that the **–**CF_3_ group can reduce the HOMO**–**LUMO energy gap for the open and closed isomers. As expected, their cyclization quantum yields (ϕ_o−c_) are much higher than their respective cycloreversion quantum yields (ϕ_c−o_), which is in accordance with other reported photochromic dithienylethenes (Li et al., 2019b,d,e). Moreover, ϕ_o−c_ and ϕ_c−o_ of dithienylethene **3**, with the OCH_3_ group, are higher compared with those of **1** and **2**, for example, ϕ_o−c_ = 0.34, ϕ_c−o_ = 0.0084 for **3**, ϕ_o−c_ = 0.13, ϕ_c−o_ = 0.0074 for **1**, ϕ_o−c_ = 0.25, ϕ_c−o_ = 0.0079 for **2**. Accordingly, **2** and **3** display much better photochromic properties than **1** without substitution.

### AIE Properties of Dithienylethenes 1–3

**Figure 2:**
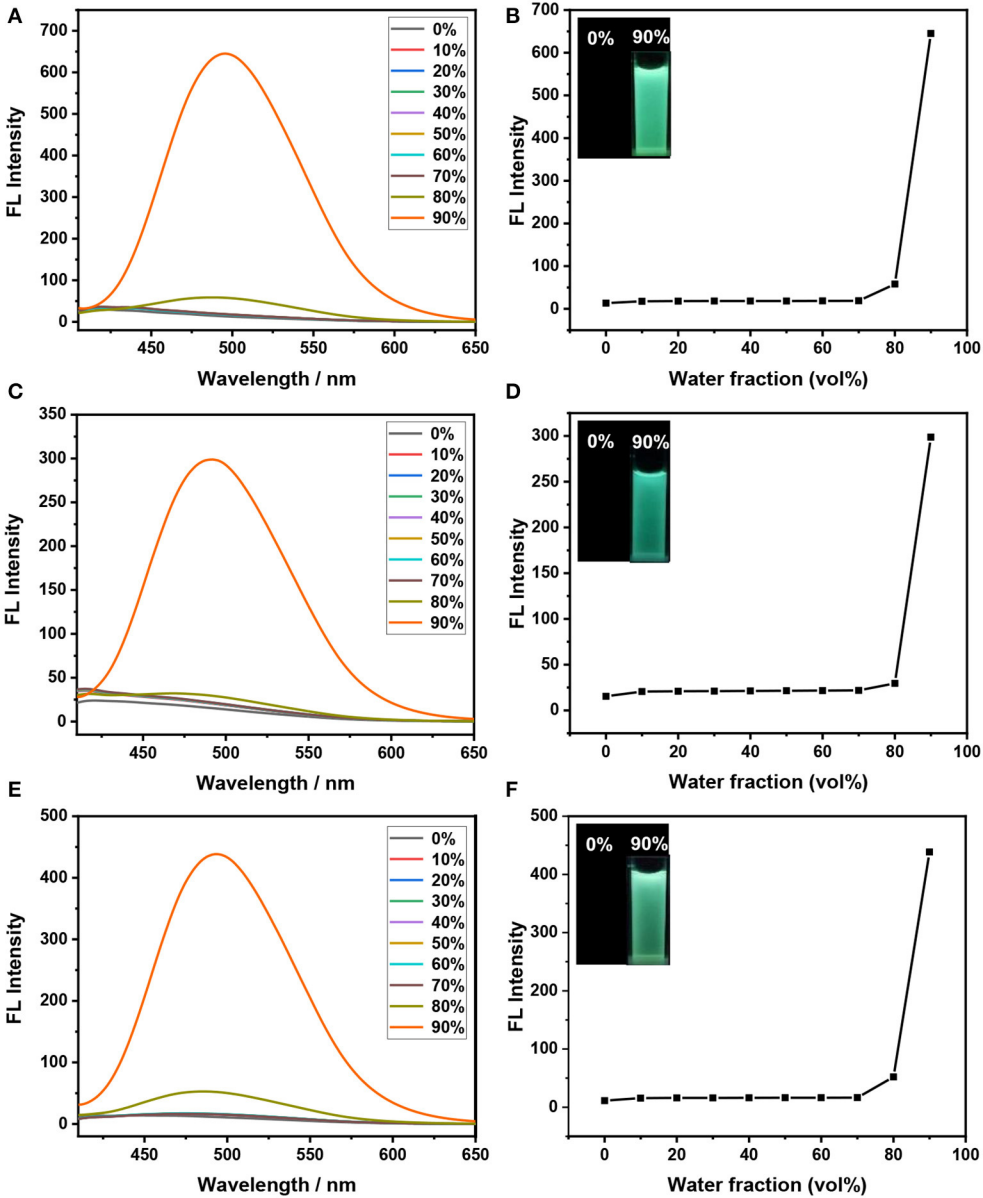
Fluorescence spectra of dithienylethenes **1–3** in different H_2_O/THF (v/v)-mixed solutions (2 × 10^−5^ mol/L) **(A,C,E)**; the dependence of the fluorescence emission intensity on the water fraction (*f*_*w*_) (Insert: photographs of dithienylethenes **1–3** in 0% and 90% water solutions under 365 nm UV light **(B,D,F)**, **(A,B)** for dithienylethene **1**; **(C,D)** for dithienylethene **2**; **(E,F)** for dithienylethene **3**.

Subsequently, the AIE properties of these triphenylethene-functionalized dithienylethenes (**1**–**3**) are explored before irradiation with UV light at 254 nm. As illustrated in Figure 2A, the open form **1 (o)** displays almost no emission in pure THF solution, meaning the three phenyl rings o nTriPE moieties can effectively dissipate the excited-state energy via intramolecular rotations. The emission spectra remain almost constant when the water fraction (*f*_*w*_) gradually increased from 0 to 70%. As *f*_*w*_ further increases, the fluorescent emission intensity at 495 nm is enhanced and reaches its maximum when *f*_*w*_is 90%, which is accompanied by green fluorescence (Figure 2B). We think the main reason for this phenomenon is that the propeller-shaped triphenylethene moieties with non-planarity prevent intermolecular π-π stacking interactions in the aggregate state, and thus blocking the non-radiative decay channels. Similar fluorescence enhancement for dithienylethenes **2** and **3** is observed with the water fraction increased from 0 to 90% (Figures 2C–F). In addition, the maximum emission wavelength of dithienylethenes **1−3** are at 495, 491, and 494 nm, respectively, which indicates that various substituents seem to slightly impact the emission of triPE moieties, which may be due to the longer distance between the substituent group and the TriPE fragment. Therefore, these results imply that all the dithienylethenes display obvious AIE properties in the mixture of THF/H_2_O.

**Figure 3:**
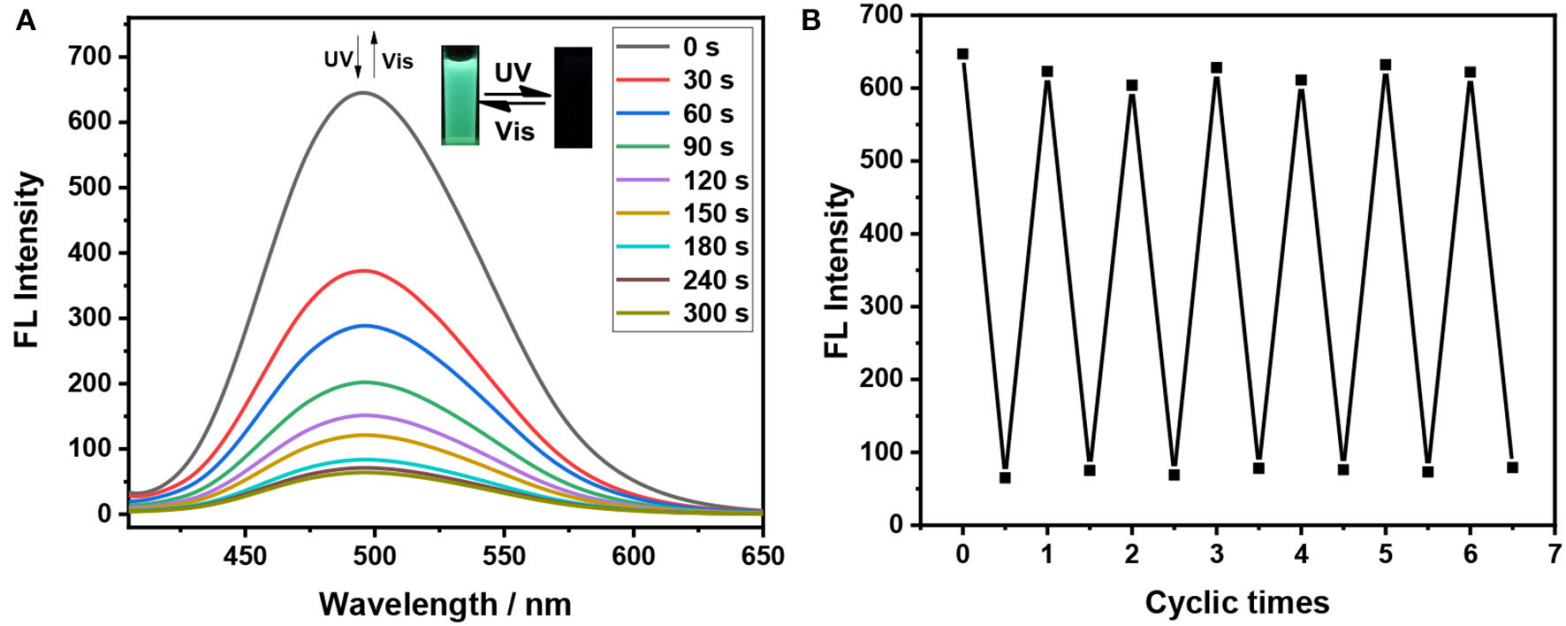
Fluorescence spectra changes of dithienylethene **1** in the mixtures of H_2_O/THF (*f*_*w*_ = 90%) (2 × 10^−5^ mol/L) upon alternating irradiation with UV light (0–300 s for the fluorescent-off state, irradiation interval: 30 s) at 254 nm and visible light at >402 nm (0–450 s for the fluorescent-on state, irradiation interval: 50), **(A)** (Inset) Corresponding fluorescent color changes upon photoirradiation in the powder state; reversible fluorescence switching for **1** in the mixtures of H_2_O/THF (*f*_*w*_ = 90%) (2 × 10^−5^ mol/L), measured at 495 nm upon alternating irradiation with UV light at 254 nm and visible light at >402 nm **(B)**.

As shown in Figure 1, the broad absorption peaks for the closed form **1(c)−3(c)** are at the regions of 417**–**650, 440**–**68, and 435**–**662 nm, respectively. Meanwhile, the maximum emission wavelength of these dithienylethenes is at 495, 491, and 494 nm in the mixtures of H_2_O/THF (*f*_*w*_ = 90%), respectively (Figure 2), which are overlapped with the absorption peaks for closed isomers. Thus, the emission may be quenched for the energy transfer from the excited TriPE segment to the ring-closed dithienylethene skeleton (Kawai et al., 2001; Wong et al., 2017). As we speculated, upon irradiation with 254 nm UV light, the emission intensity at 495 nm for **1** in the mixtures of H_2_O/THF (*f*_*w*_ = 90%) gradually decreases, which is accompanied by obvious fading of the green fluorescence due to the formation of the corresponding closed isomer (Figure 3A). The original emission could be restored upon irradiation with >402 nm visible light. Furthermore, good fatigue resistance in the mixtures of H_2_O/THF (*f*_*w*_ = 90%) is also observed from the view of the fluorescence-switching cycle (Figure 3B). Thus, these compounds display an excellent fluorescent-switching behavior when irradiated with UV/Vis light in the aggregated state.

### Fluorescent-Switching Behaviors in the Solid State

**Figure 4:**
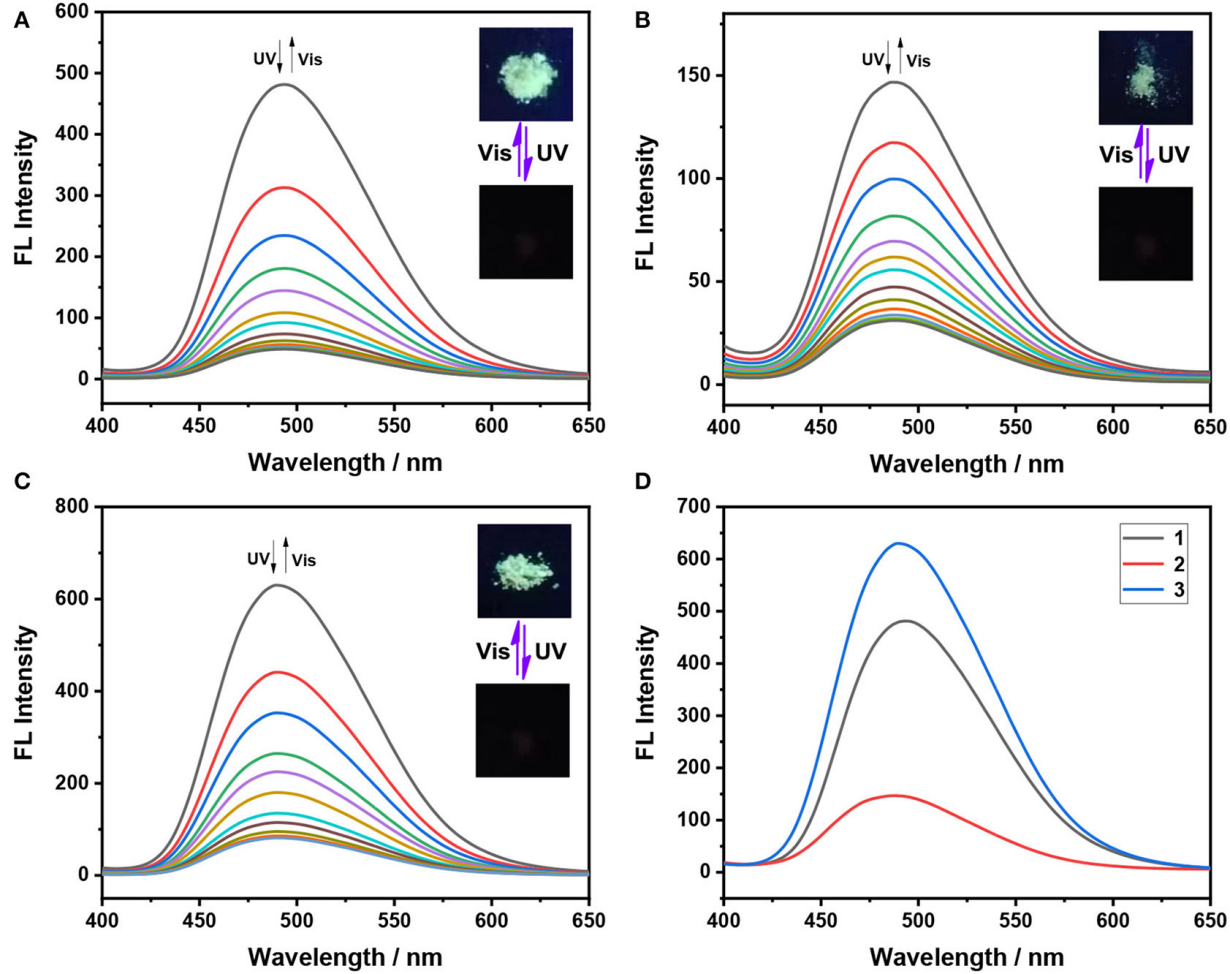
Fluorescence spectra changes of dithienylethenes **1–3** in the solid state upon alternating irradiation with UV light at 365 nm and visible light at >402 nm, **(A)** spectral changes for **1** (0–800 s for the fluorescent-off state, irradiation interval: 40 s; 0–1,200 s for the fluorescent-on state, irradiation interval: 60 s); **(B)** spectral changes for **2** (0–560 s for the fluorescent-off state, irradiation interval: 40 s; 0–960 s for the fluorescent-on state, irradiation interval: 60 s); **(C)** spectral changes for **3** (0–480 s for the fluorescent-off state, irradiation interval: 40 s; 0–720 s for the fluorescent-on state, irradiation interval: 60 s); **(D)** fluorescence emission spectra of ring-open isomers **1o−3o** in the solid state. (Inset) Corresponding fluorescent color changes upon photoirradiation in the powder state.

For many applications, especially those that facilitate device integration, the fluorescence switch is ideal for being able to trigger effectively on solid or solid supports (Cheng et al., 2015; Lehr et al., 2015). Next, we further investigate the fluorescent-switching behaviors of these dithienylethenes in the powder state. As displayed in Figure 4A, Table 1, **1 (o)** in the powder state emits strong green fluorescence at λ_em_ = 493 nm, which implies a negligible hypochromatic shift, compared to that in the aggregated state (495 nm). Furthermore, **3** with OCH_3_ group displays the strongest emission intensity than those of its analogs **1** and **2**. Similar to that, for the aggregated state, dithienylethene **1** exhibits the efficient- and reversible-fluorescence “on**–**off” process in the powder state upon alternating irradiation with 365 nm UV light and visible light at >402 nm. Similar fluorescent-switching behaviors are also observed when the powder state of **2** and **3** is exposed to 254 nm UV light and >402 nm visible light, respectively, as illustrated in Figures 4B–D. Thus, they can be utilized as a novel fluorescence switch integrated with smart, solid-state optoelectronic materials.

### Theoretical Calculations

**Figure 5:**
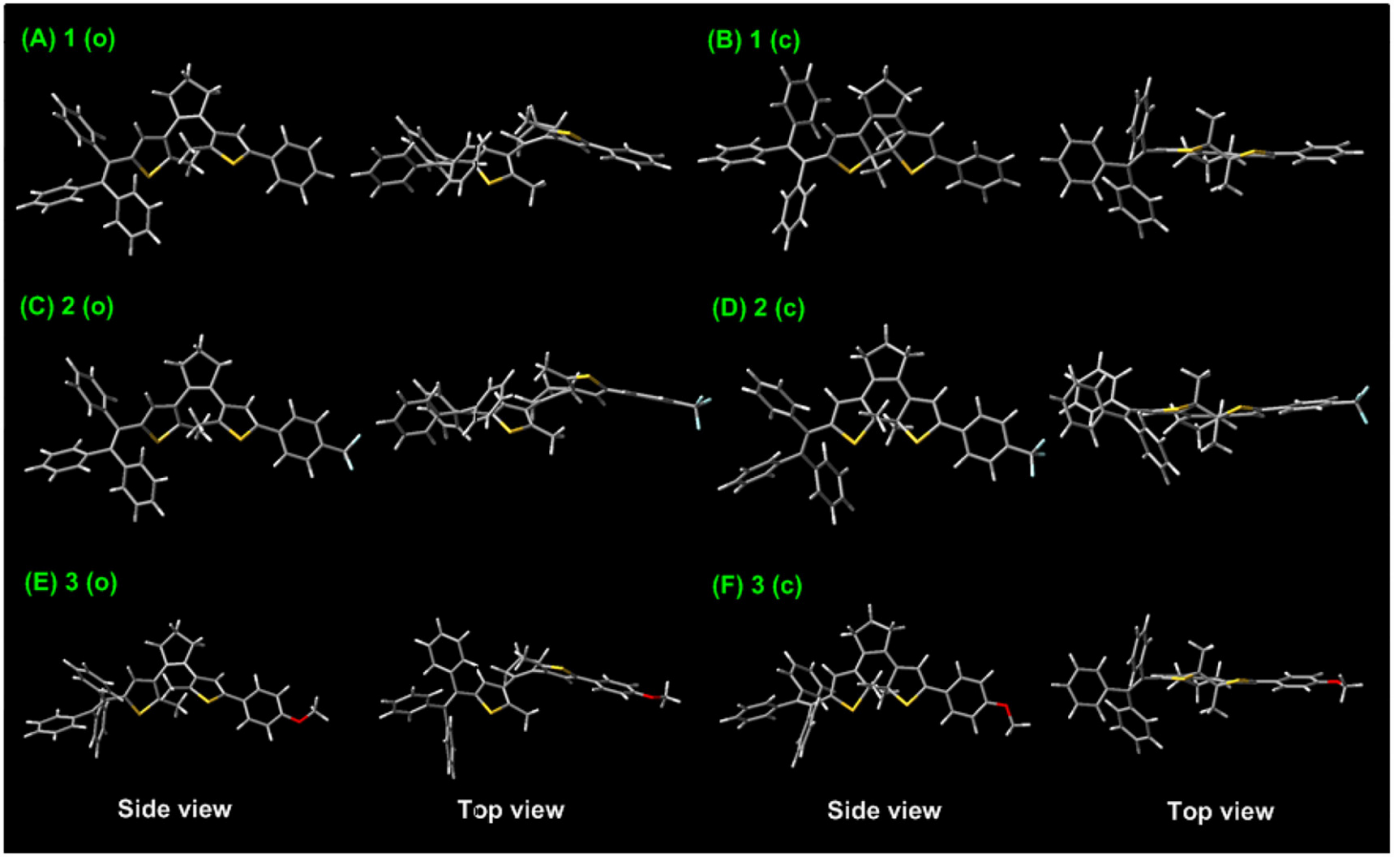
Optimized ground-state geometry of dithienylethenes **1–3** based on DFT calculations at the B3LYP/6-31G* level by using the Gaussian 09 program, **(A)** ring-open isomer 1 (o); **(B)** ring-closed isomer 1 (c); **(C)** ring-open isomer 2 (o); **(D)** ring-closed isomer 2 (c); **(E)** ring-open isomer 3 (o); **(F)** ring-closed isomer 3 (c).

**Figure 6:**
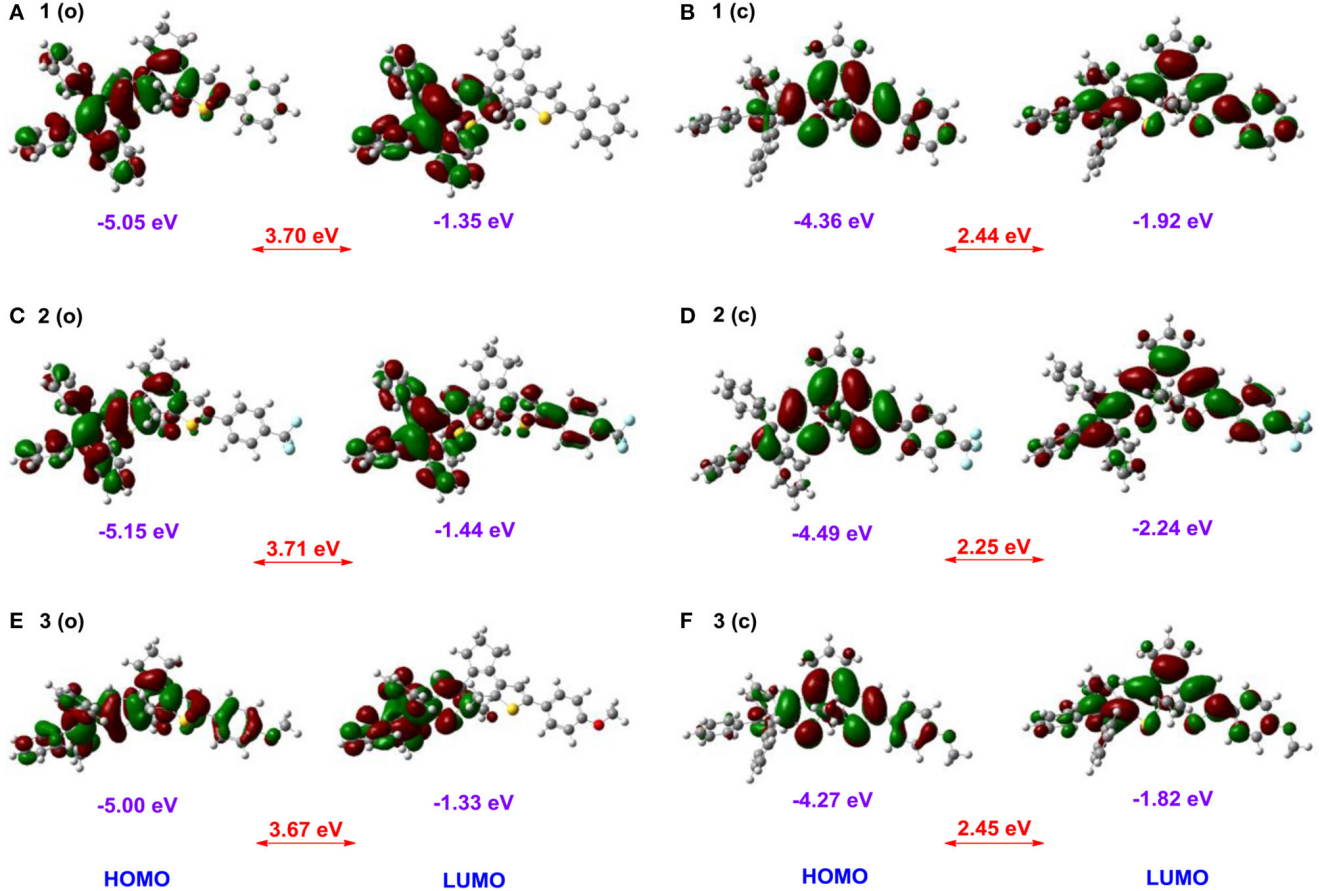
Frontier molecular orbital profiles of dithienylethenes **1–3** based on DFT calculations at the B3LYP/6-31G* level by using the Gaussian 09 program, **(A)** ring-open isomer 1 (o); **(B)** ring-closed isomer 1 (c); **(C)** ring-open isomer 2 (o); **(D)** ring-closed isomer 2 (c); **(E)** ring-open isomer 3 (o); **(F)** ring-closed isomer 3 (c).

To further gain an insight into the relationships between the electronic properties and photoreactivity of **1–3**, their ground-state geometry and electron density are calculated by density functional theory (DFT) in Gaussian 09 B3LYP/6-31G^*^ level (Ditchfield, 1971; Becke, 1993; Frisch et al., 2009). As illustrated in Figure 5A, the energy-minimized structure of **1 (o)** displays a classical antiparallel conformation, in which triphenylethene moieties attached to the adjacent thiophene group show a propeller configuration. Moreover, the HOMO orbital energy of **1 (o)** is localized around the triPE and DTE moieties, while its LUMO is mainly distributed over the triPE group due to its poor planarity (Figure 6A). Thus, the results further confirm its AIE properties and luminescence behaviors in the solid state in the experiments, which is mainly because the propeller-shaped triphenylethene moieties with non-planarity can block intermolecular π**–**π stacking interactions in the aggregate state and the solid state. In addition to the triPE group, the closed isomer **1 (c)** presents an almost planar conjugated structure (Figure 5B), in which the HOMO is mainly distributed in the DTE center while its LUMO is nearly on the whole molecular skeleton (Figure 6B). As expected, compared with **1 (o)** (3.7 eV), **1 (c)** displayed a narrower energy band gap (2.44 eV) due to the extended π-conjugation. For the CF_3_/OCH_3_-substituted dithienylethenes **2** and **3**, analogically optimized structures and electron distributions for open and closed forms are observed (Figures 5C–F, 6C–F). Thus, the DFT calculations further validate the above experimental results.

## Conclusions

In summary, we successfully have developed three novel triphenylethene-functionalized dithienylethenes by introducing triphenylethene moieties at the termini of dithienylethene unit, in which the triPE group functions as an AIE active fragment. They display good photochromic behaviors with excellent fatigue resistance upon irradiation with UV (254 nm) or visible light (>402 nm) in THF solution. And it has been found out that different substituent groups have a slight effect on their photochromic properties, mainly including the absorption maximum and quantum yields of cyclization and cycloreversion reactions. Moreover, these compounds exhibit AIE properties and luminescence behaviors in the solid state before irradiation with UV light. Upon alternating irradiation with UV/visible light, they display effective fluorescent-switching behaviors in the aggregated state and the solid state. The experimental results have been validated by the DFT calculations. Thus, they can be utilized as novel fluorescence switches for potential application in the fields of optoelectronics and photopharmacology.

## Data Availability Statement

The original contributions presented in the study are included in the article/Supplementary Material, further inquiries can be directed to the corresponding author/s.

## Author Contributions

HZhu, CL, and XG performed the synthesis experiments. XH, XZ, and LS conducted the properties. HZha, Y-PZ, LL, and ZL designed the experiments. HZha, Y-PZ, and ZL interpreted the data and wrote the paper. All the authors contributed to the article and approved the submitted version.

## Conflict of Interest

The authors declare that the research was conducted in the absence of any commercial or financial relationships that could be construed as a potential conflict of interest.
