# Supplementary material for: A Solid-State Fluorescence Switch Based on Triphenylethene-Functionalized Dithienylethene With Aggregation-Induced Emission
Source: Front Chem. 2021 Apr 28;9:665880. doi: 10.3389/fchem.2021.665880 (PMC8113874; doi:10.3389/fchem.2021.665880)
Supplement: Supplementary file 1 [file Data_Sheet_1.docx]

***Supporting Information for***

**A Solid-State Fluorescence Switch Based on Triphenylethene Functionalized Dithienylethene with Aggregation-Induced Emission**

Haining Zhang^1^, Xiaoxiao Hu^1^, Huijuan Zhu^1^, Limin Shen^1^, Congmin Liu^1^, Xiaoman Zhang^1^, Xinyu Gao^1^, Lingmei Li^1^, Yan-Ping Zhu^2*^ and Ziyong Li^1*^

*^1^ Luoyang Key Laboratory of Organic Functional Molecules, College of Food and Drug, Luoyang Normal University, Luoyang, 471934,* *P. R. China*

*^2^ School of Pharmacy, Key Laboratory of Molecular Pharmacology and Drug Evaluation, Ministry of Education, Collaborative Innovation Center of Advanced Drug Delivery System and Biotech Drugs in Universities of Shandong,* *Yantai University, Shandong, Yantai, 264005, P. R. China.*

Corresponding author E-mail: [liziyong@mails.ccnu.edu.cn](mailto:liziyong@mails.ccnu.edu.cn); [chemzyp@foxmail.com](mailto:chemzyp@foxmail.com)

**FIGURE S1.** Fatigue resistance of **1** upon alternating irradiation with 254 nm UV light and visible light at >402 nm over six cycles in DMSO (2.0 × 10^-5^ mol/L).

**FIGURE S2.** Fatigue resistance of **2** upon alternating irradiation with 254 nm UV light and visible light at >402 nm over six cycles in DMSO (2.0 × 10^-5^ mol/L).

**FIGURE S3.** Fatigue resistance of **3** upon alternating irradiation with 254 nm UV light and visible light at >402 nm over six cycles in DMSO (2.0 × 10^-5^ mol/L).

**Appendix: NMR and Mass spectra**


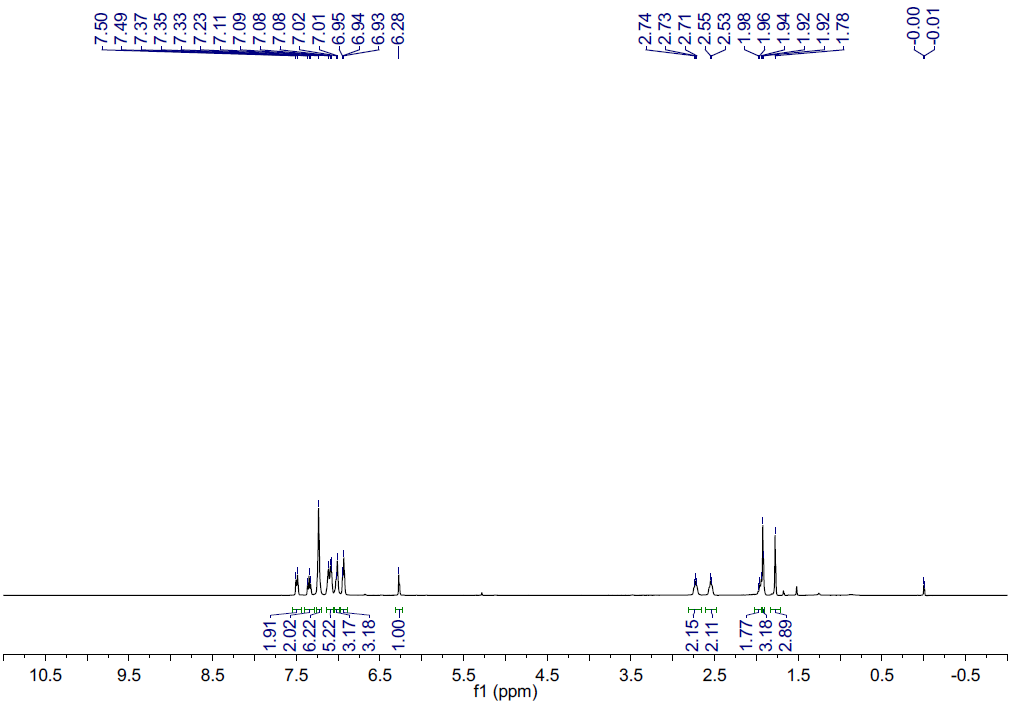


**FIGURE S4**. 400 MHz ^1^H NMR spectrum of dithienylethene **1** in CDCl_3_ at room temperature.


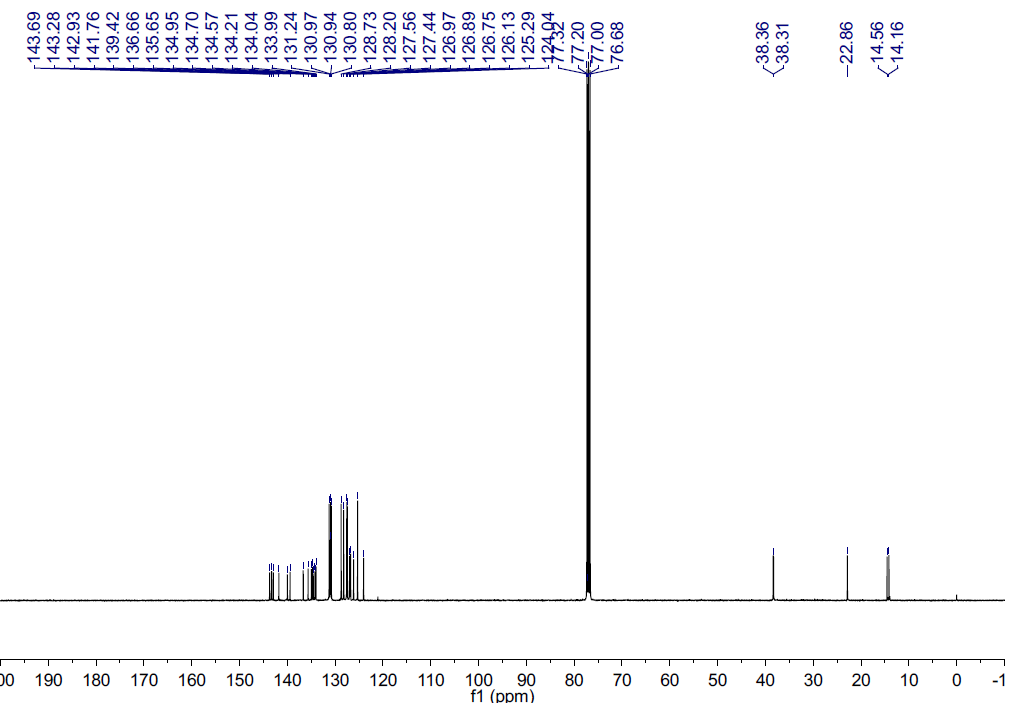


**FIGURE S5**. 100 MHz ^13^C NMR spectrum of dithienylethene **1** in CDCl_3_ at room temperature.


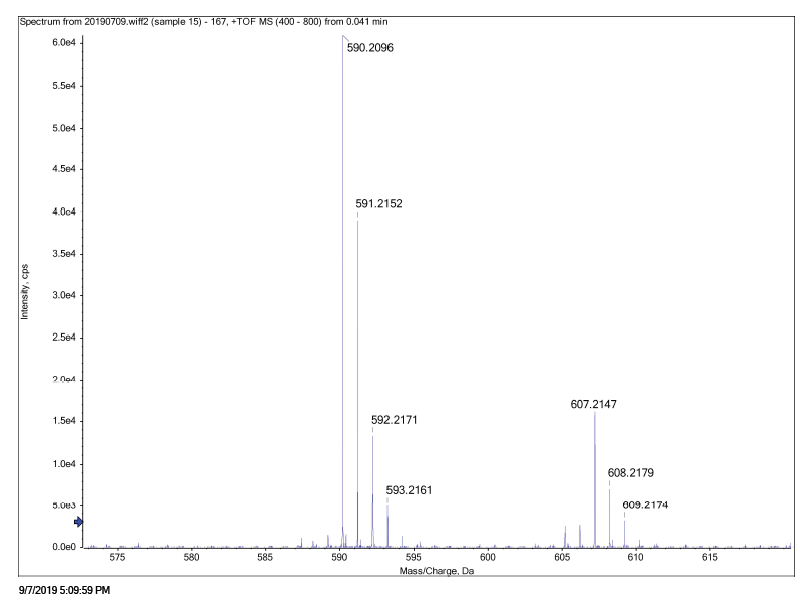


**FIGURE S6**. HRMS of dithienylethene **1** at room temperature.


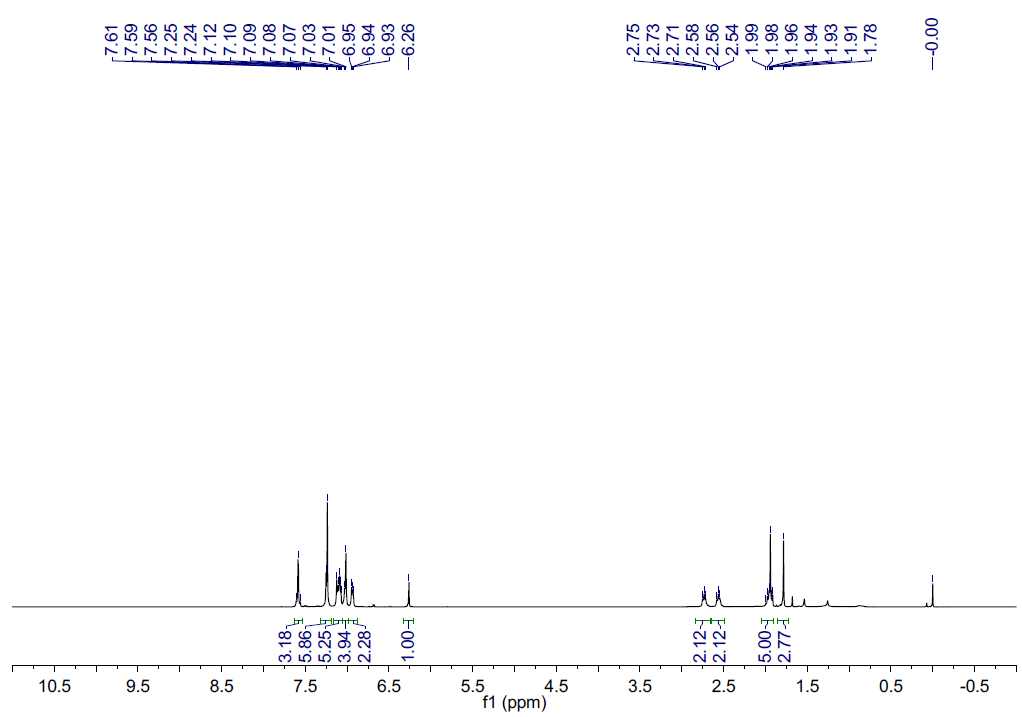


**FIGURE S7**. 400 MHz ^1^H NMR spectrum of dithienylethene **2** in CDCl_3_ at room temperature.


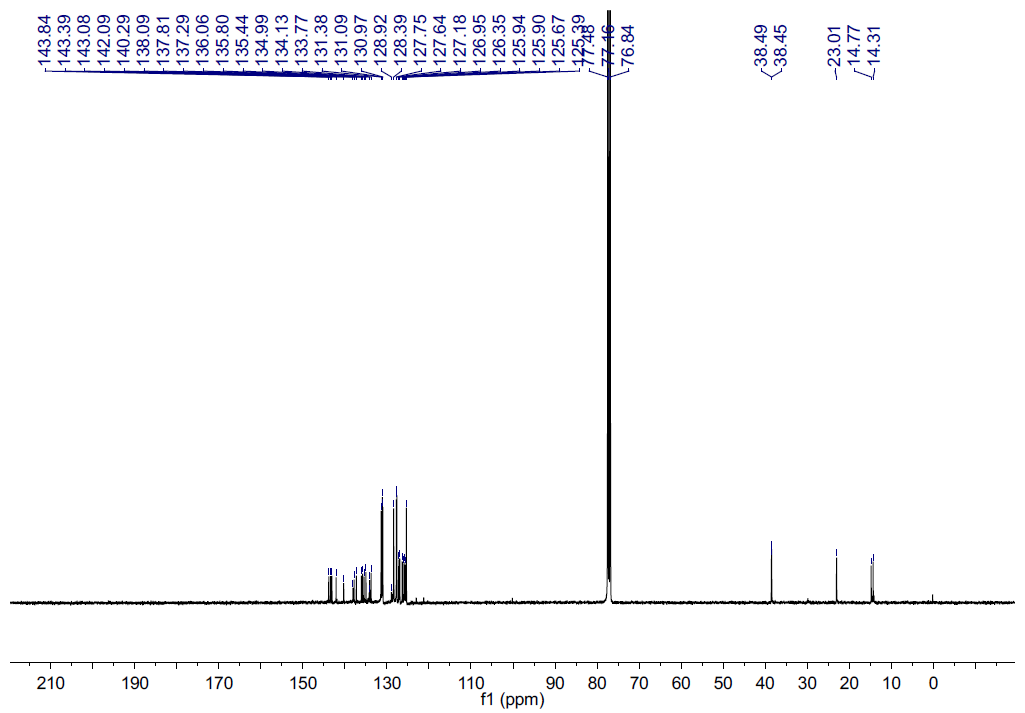


**FIGURE S8**. 100 MHz ^13^C NMR spectrum of dithienylethene **2** in CDCl_3_ at room temperature.


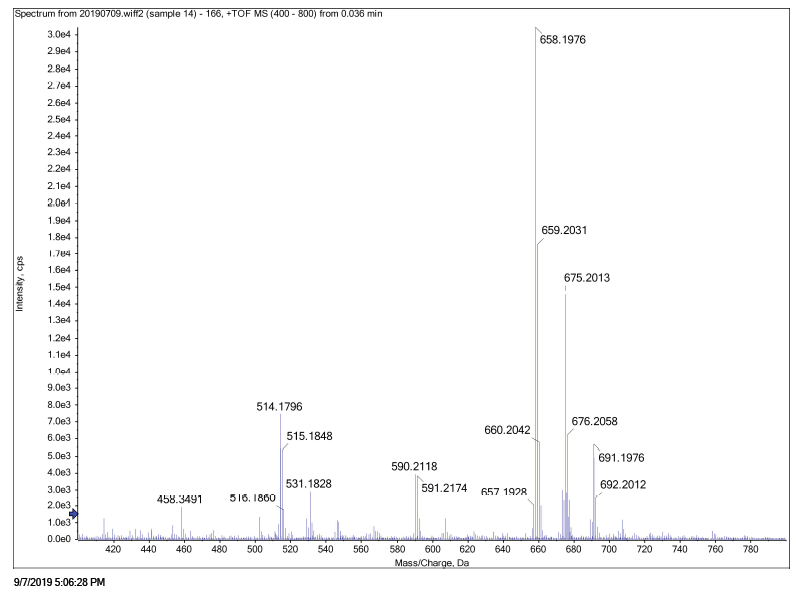


**FIGURE S9**. HRMS of dithienylethene **2** at room temperature.


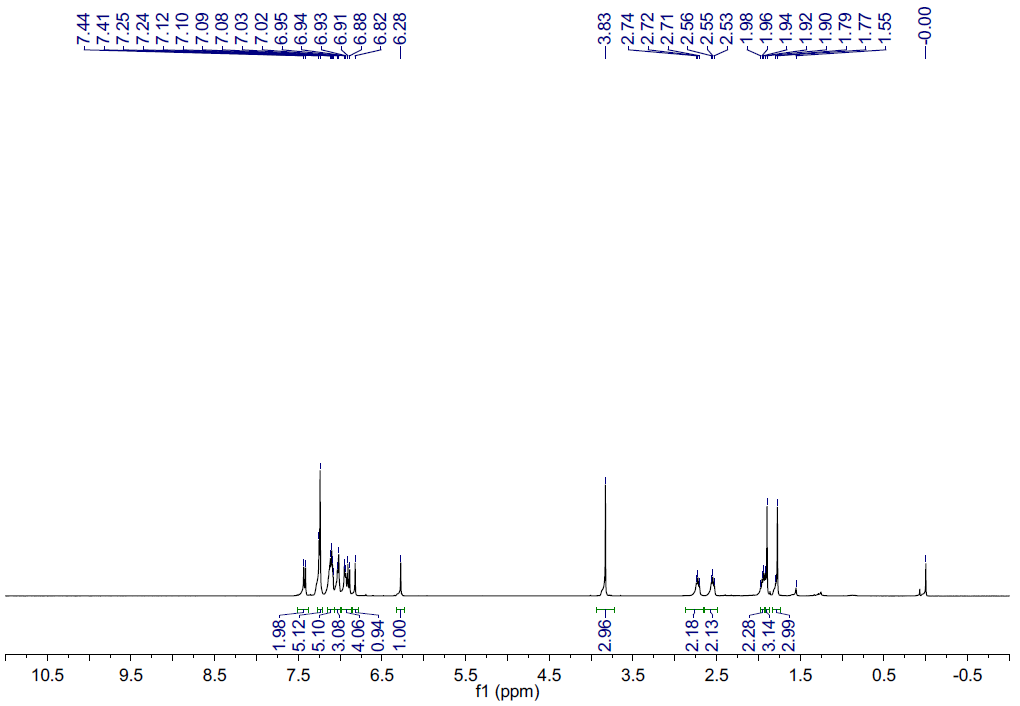


**FIGURE S10**. 400 MHz ^1^H NMR spectrum of dithienylethene **3** in CDCl_3_ at room temperature.


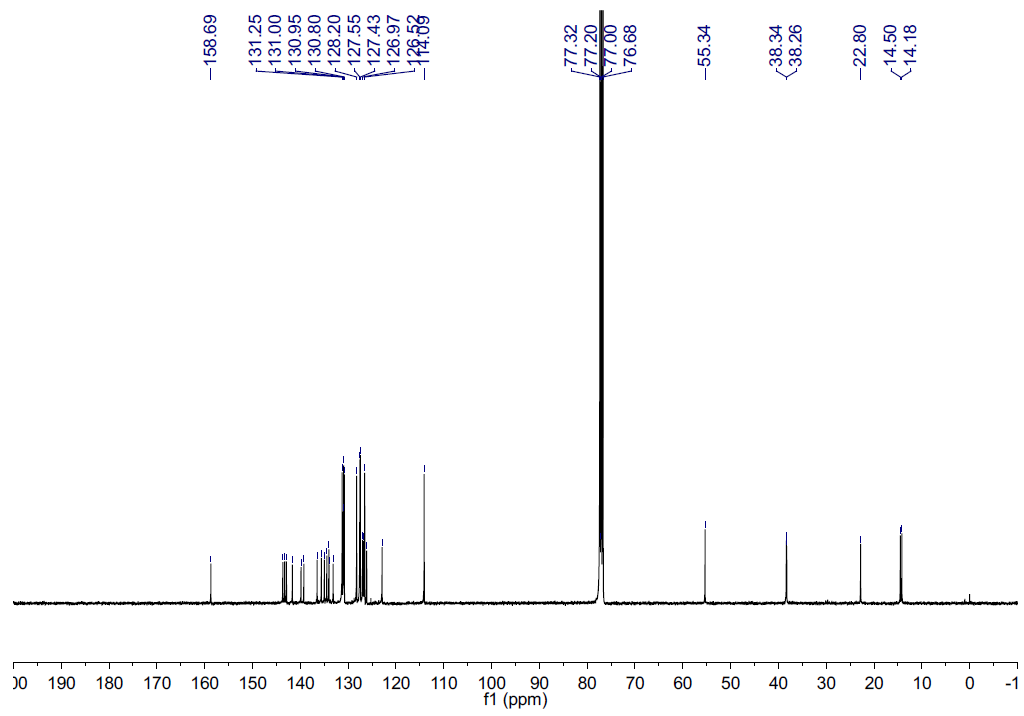


**FIGURE S11**. 100 MHz ^13^C NMR spectrum of dithienylethene **3** in CDCl_3_ at room temperature.


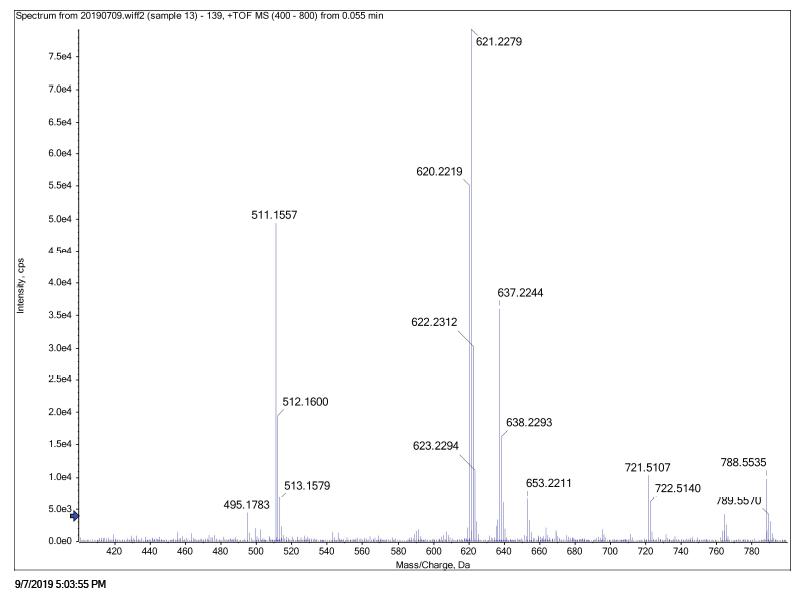


**FIGURE S12**. HRMS of dithienylethene **3** at room temperature.
